# Dysfunctional vestibular system causes a blood pressure drop in astronauts returning from space

**DOI:** 10.1038/srep17627

**Published:** 2015-12-16

**Authors:** Emma Hallgren, Pierre-François Migeotte, Ludmila Kornilova, Quentin Delière, Erik Fransen, Dmitrii Glukhikh, Steven T. Moore, Gilles Clément, André Diedrich, Hamish MacDougall, Floris L. Wuyts

**Affiliations:** 1 Antwerp University Research center for Equilibrium and Aerospace, Dept. of Biomedical Physics, University of Antwerp, Belgium; 2 Université libre de Bruxelles & Erasmus Hospital, Department of Cardiology, Laboratory of Physics and Physiology, Brussels, Belgium; 3 Laboratory of Vestibular Physiology, Institute of Biomedical Problems of the Russian academy of sciences, Moscow, Russia; 4 StatUa center for statistics, University of Antwerp, Antwerp, Belgium; 5 Human Aerospace Laboratory, Icahn School of Medicine at Mount Sinai, New York City, USA; 6 Lyon Neuroscience Research Center, IMPACT Team, University of Lyon, France; 7 Autonomic Dysfunction Center, Vanderbilt University School of Medicine, Nashville, USA; 8 Sydney Human Factors Research, School of Psychology, University of Sydney, Australia.

## Abstract

It is a challenge for the human body to maintain stable blood pressure while standing. The body’s failure to do so can lead to dizziness or even fainting. For decades it has been postulated that the vestibular organ can prevent a drop in pressure during a position change – supposedly mediated by reflexes to the cardiovascular system. We show – for the first time – a significant correlation between decreased functionality of the vestibular otolith system and a decrease in the mean arterial pressure when a person stands up. Until now, no experiments on Earth could selectively suppress both otolith systems; astronauts returning from space are a unique group of subjects in this regard. Their otolith systems are being temporarily disturbed and at the same time they often suffer from blood pressure instability. In our study, we observed the functioning of both the otolith and the cardiovascular system of the astronauts before and after spaceflight. Our finding indicates that an intact otolith system plays an important role in preventing blood pressure instability during orthostatic challenges. Our finding not only has important implications for human space exploration; they may also improve the treatment of unstable blood pressure here on Earth.

Movements and position changes are followed by a series of physiological changes in the human body. One of them is the maintenance of stable blood pressure during a position change. However, its underlying mechanism is still not fully understood. It is crucial to keep a sufficient blood flow to the brain at all times. The inability to do so is called Orthostatic Intolerance (OI), which causes a drop in blood pressure. The main cause of orthostatic hypotension is today attributed to an excessive fall of cardiac output or a defect vasoconstrictor mechanism^1^. Orthostatic intolerance can lead to dizziness and even evoke syncope, that is, fainting. A blood pressure drop upon standing is typically linked to the dispensability of the arteries in the lower limbs, as well as failure of the arteries to constrict timeously. Consequently, blood pools in the lower parts of the body. Thus, the functional changes accompanying OI can be understood as a temporal mismatch between cardiac output and vascular resistance^2^. Conversely, a successful constriction leads to a redistribution of blood and an increase in blood pressure. The symptoms of OI can cause serious problems and have a strong impact on the quality of life of the affected person.

The sympathetic nervous system (SNS), which is responsible for cardiac regulation, has been pointed out as a key factor in blood pressure control^3^,^4^. During postural changes a range of feedback mechanisms serve to increase firing in the sympathetic nerves, which, among other effects, leads to an increased blood pressure. One of the reflexes that contributes to these mechanisms is thought to originate in the vestibular organs.

The vestibular organs, located in the inner ears, sense rotations by means of three semi-circular canals, and they sense linear accelerations, including gravity, by means of the otoliths. Together with information provided by the visual and proprioceptive system, the vestibular organs ensure gaze stabilization and balance as well as orientation and navigation. This task is so fundamental to life on Earth that most animals have a highly specialized vestibular system, the mammalian one being very similar across many species.

Early animal studies suggest that the vestibular system plays an important role in the activation of the SNS^3^,^5^,^6^,^7^,^8^,^9^,^10^,^11^,^12^,^13^,^14^. By selective natural and electrical vestibular stimulation, an increase of the activity in the SNS and of the blood pressure has been measured. Those results strengthen the evidence for a presence of a vestibulo-sympathetic reflex (VSR) in animals. This reflex links a stimulation of the vestibular system with an activation of the cardiovascular system.

Vestibular signals have been shown to affect mainly the sympathetic outflow to the blood vessels. In fact, all the activated nerves contain vasoconstrictor efferents^15^. The VSR is therefore thought to be particularly important in the prevention of a drop in blood pressure and of OI because the VSR can be elicited at the first sensing of motion. The otoliths detect a position change with respect to gravity within milliseconds and play a key role in the activation of the VSR. Altered sympathetic nerve activity has been registered upon their stimulation with a short latency (660 ms), suggesting that this may be one of the earliest mechanisms to sustain blood pressure upon standing up^16^,^17^. The latency in animals has been shown to be as short as 50–100 ms^3^. Based on the information from the otoliths, the activation of the SNS increases pre-emptively before an actual drop in blood pressure is detected, constituting a feed-forward mechanism. For instance, the baroreceptors are not activated until an actual blood pressure drop is measured in the carotid arteries. Hence, they initiate a feed-backward mechanism with a latency of 1.2–1.4 s^17^.

A few later studies show that the same theory might be applicable to humans^3^,^5^,^16^,^17^,^18^,^19^,^20^. However, all the studies on this phenomenon have so far focused on an activation of the otoliths^1^. None of these experiments allows a selective suppression of the otolith reflex. Thus, little evidence exists for a decreased VSR in humans as a result of otolith deficiency. However, isolation of the otolith system is essential to studying a hampered VSR. We aim to elucidate the VSR in the case of a so-called ‘deconditioned otolith system’, which means that the functioning of the system is decreased. Such deconditioned otoliths can uniquely be found in astronauts returning from a spaceflight.

A majority of the astronauts returning from a long-term exposure to microgravity suffer from OI during the first days after their return^21^,^22^. Since the beginning of manned spaceflight, symptoms such as dizziness, postural control problems and even cases of syncope have been reported. It has been hypothesized that the decreased cardiovascular response is due to the prolonged weightlessness experienced during a spaceflight^23^, presumably caused by the adaptation of autonomic control and the deconditioning of cardiac regulation mechanisms^24^,^25^. A number of countermeasures to fluid shift and OI have been tried out during the history of spaceflight^26^, but so far with little success for the returning space travellers suffering from OI. In space, the cardiovascular system is no longer exposed to the transitions generated by a position change, which stimulates the reflex needed to counteract gravity.

Similarly to the cardiovascular system, the otoliths are also forced, during spaceflight, to adapt to a situation where there is no longer a preferred direction given by the acceleration of gravity^27^. For some years now, it has been shown that the otolith system, due to the absence of perceived gravity, is suppressed in returning astronauts^22^. In addition, results from the Neurolab shuttle mission demonstrated that the otolith function was maintained during and after flight in payload crewmembers exposed to in-flight 1-g centripetal acceleration during centrifugation (‘artificial gravity’). Non-centrifuged crewmembers however exhibited clear signs of OI, whereas astronauts exposed to ‘artificial gravity’ did not^25^.

In view of this phenomenon, we hypothesized that the otoliths play a key role in OI, affecting the astronauts during their first days after spaceflight. The goal of our study has been to investigate if a suppressed otolith system affects the ability to regulate blood pressure upon standing, on return from space. In this way we could study the link between the vestibular and the cardiovascular system to elucidate the VSR.

In order to study the VSR, we needed to evaluate brainstem-mediated reflexes that were based on information from the vestibular system, in particular ocular counter-rolling (OCR). OCR is an otolith-mediated reflex that serves as a compensatory eye movement. It is generated when we tilt our head sideways (head changes with respect to the gravitational vector), turn around a corner or undergo off-axis centrifugation^28^. During centrifugation, the otolith system perceives a tilt caused by the combination of centripetal forces and gravity and therefore generates OCR.

Similarly, the cardiovascular system can be quantified by specific parameters. Mean arterial pressure (MAP) is one of the most important parameters resulting from the cardiac and cardiovascular control mechanisms. Indeed, MAP is considered to be the perfusion pressure seen by the organs and is directly influenced by stroke volume (SV), heart rate (HR), systemic vascular resistance (SVR) and central venous pressure (CVP):


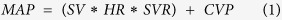


As a result, MAP can be considered the output of the overall cardiovascular system. The goal is to maintain an appropriate perfusion of all organs via action on the cardiac function (HR, SV) and the vascular system (SVR, CVP). However, HR and heart rate variability (HRV) are also markers of the autonomic control of the cardiovascular system.

Our primary outcomes in this study are the values of the OCR, the MAP and their correlation.

## Methodology

We conducted our experiments in the **G**agarin **C**osmonaut **T**raining **C**entre (Star City) near Moscow, Russia. A group of 12 male (47 + /−5 years, 78.3 + /−5.8 kg, 179.3 + /−4.0 cm) cosmonauts from the Russian Space Agency (Roscosmos) and one from European Space Agency (ESA) (all denoted as astronauts) took part in the study. The astronauts were tested before and after a 6-month stay in the International Space Station (ISS). The preflight data were based on two baseline experiments that were conducted on average 66 (SD = 26) days before launch. The postflight data consist of one experiment performed in the early days (3.8, SD = 1.7 days) after return back to earth, and another experiment performed 9–10 days (9.6, SD = 0.51) after return. The two experiments will be denoted as “early postflight” and “late postflight”. Due to medical and administrative issues we were not able to test all the astronauts on the same day after return.

To quantify the effect of microgravity on the vestibular otolith system and on the cardiovascular system respectively, two separate tests were performed. During the first test the astronaut was subjected to centrifugation to induce the otolith parameter Ocular Counter-Rolling (OCR). Similarly to eliciting an otolith reflex by centrifugation, the cardiovascular system control mechanism can be elicited by an operational tilt test. The second test was meant to put the cardiovascular system under orthostatic stress and evaluate its reaction. The responses evaluated were MAP, HR and HRV. Moreover, the autonomic control of the HR and the reactivity of the cardiovascular system were evaluated with the analysis of total power of HRV, as well as LF (low frequency) and HF (high frequency) components separately. The latter is also known as respiratory sinus arrhythmia (RSA), a marker of vagal modulation of HR. The RR-interval (RRI), used in HRV computation, was also computed as:


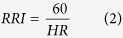


As RRI is used for the analysis of HRV^29^, RRI is presented instead of HR in the figures. However for the argumentation and discussion we still make use of the more standard HR.

### VVIS – evaluation of the otolith system

**Figure 1:**
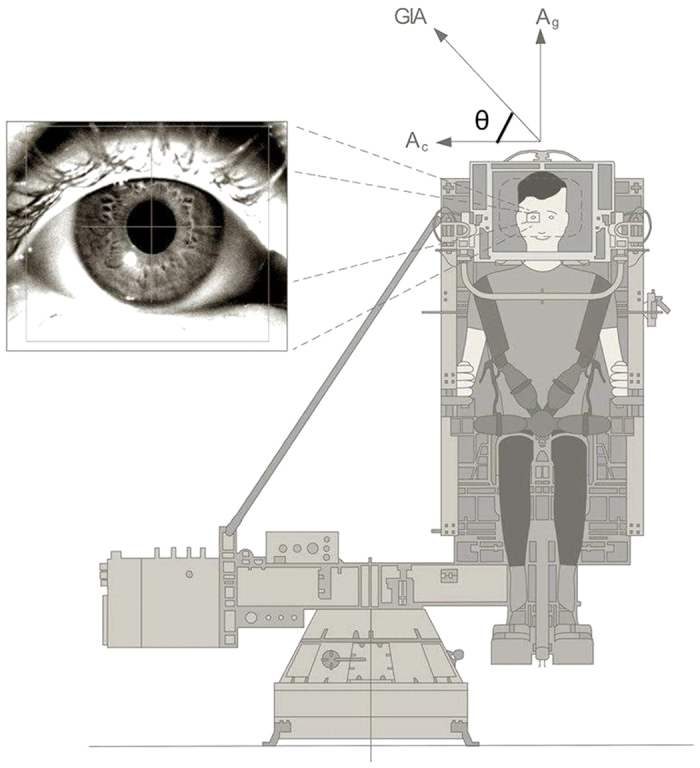
VVIS chair. The drawing shows a model of the centrifuge we used to induce OCR. During off-axes centrifugation, the net linear acceleration stimulating the otoliths is the (vector) sum (GIA) of the centripetal force and gravity. (See vectors in the figure) The GIA will be interpreted as the vertical, with the consequence that the subject experiences a sensation of tilt. The OCR, a torsion of the eye, was recorded using three-dimensional infrared video-oculography. As a second step we analysed the recording of the eye movement for every experiment to calculate the OCR in degrees.

During the first experiment, the astronaut was installed in the **V**isual and **V**estibular **I**nvestigation **S**ystem (VVIS), a small centrifuge (rotation chair, see Fig. 1) build for the Neurolab shuttle mission. The astronaut was securely fixed in the chair and head movements were restricted. The entire room was darkened to avoid visual motion feedback during rotation. The centrifuge allowed earth vertical rotation on a fixed distance of 0.5 m from the axes of rotation. In front of the astronaut a screen was placed on which visual targets were present during parts of the experiments. After calibration of the video-goggles and a recording of a baseline the astronaut was subjected to the 1 g for 5 minutes in counter clockwise (CCW) and 5 minutes in clockwise (CW) direction. The subject was always facing the direction of motion. Right-ear out (REO) during CCW rotation and left-ear out (LEO) during CW. The maximum velocity of 254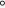/s was chosen to obtain a centripetal acceleration of 1 g outwards. Combined with gravity, such a shear force constitutes a virtual sideways tilt of 45 degrees, inducing an OCR of typically a couple of degrees.

Measurements of the OCR were taken before and during rotation according to the protocol. The first measurement was taken during stand still, the second one was taken 40 seconds after the stable phase of rotation was reached. The 40 seconds of delay was implemented to allow the cupola of the horizontal semi circular canal to return to its original position, to make sure that the measured OCR was based on contribution of the otolith system only. The difference in OCR between stand still and rotation was defined as the OCR value, 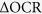
_preflight_. The preflight data was compared with the OCR recorded during the early postflight experiment, the 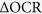
_postflight_. The difference in OCR (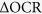), a comparison between pre- and postflight, gave us an indication of the influence of microgravity on the otoliths.

Measurements of the OCR give a reliable estimate of the otolith function^25^ and are typically done using three-dimensional infrared video-oculography^30^. The videos containing recordings of the eye movements were analyzed to calculate the torsion of the eyes, e.g. the OCR. To do this we run the files in a visual programming language. The program we used was custom made in National Instruments LabVIEW by one of the co-authors. Further statistical analyses were made in PASW Statistics (SPSS Software®, SPSS).

### Operational tilt-test –evaluation of the cardiovascular system

Right after the centrifugation we performed the second test, an operational tilt test, to study the cardiovascular system. Similar to provoking an otolith reflex by centrifugation, the cardiovascular system can be provoked by an operational tilt test. The response of the cardiovascular system was quantified with the following parameters HR, HRV, HF and MAP. During such a test the subject is exposed to orthostatic stress by inducing a passive change of position. The subject is initially positioned horizontally on the table, at one point the table is tilted to place the subject in a passive upright position. This causes concomitant orthostatic stress, high enough to create a risk of fainting. Essential during such a test is that the subject passively undergoes the position change, and no muscle pumps are involved to actively regulate blood pressure changes. The subject was tested in supine position (laying down) before putting the table in an upright position for the second measurement in standing position. In the same way as when analysing the OCR, we compared the preflight data for the cardiac and blood pressure control with the postflight data. Measurements were performed during a 60 seconds long recording. For supine position the measurement took place between the 4th and 5th minute of the protocol and for the upright position between the 9th and 10th minute during a passive tilt of 60 degrees. The difference between cardiac and blood pressure control during standing and supine position, as well between pre- and post-flight, served as a measurement of the cardiovascular function, the so-called 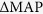, 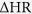, 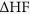, and 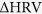. In this way we obtained, for each subject, a specific measure of the otolith function and of the cardiovascular function. For the analyses of the cardiovascular parameters, a Matlab program tailored to the needs of those experiments by one of the co-authors was used.

### Statistics

By comparing the OCR before and after spaceflight, we could study the effect of prolonged microgravity on the otolith system. Similarly, we could estimate the impact of prolonged microgravity on the cardiovascular system by assessing the reaction to the standing position. The final step was to check for correlation between the 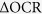 and the cardiovascular parameters (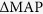, 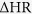, 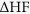, and 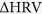). In this way we would know if there was a link between the change in otolith function and a change in the ability to control the blood pressure during a position change. Before performing any further analyses we used the Kolmogorov-Smirnov test in SPSS to test the normality of the two variables tested for correlation. In both cases the variables were normally distributed (p = 0.2). To check for correlation a Pearson correlation test was performed (in SPSS) between the OCR and the cardiovascular parameters. Using the pwr package in R, we calculated the samples sizes required to have an 80% power to detect moderate to high correlation coefficients. The current sample size (12 individuals), holds 80% power to detect a Pearson correlation coefficient of 0.72.

## Results

Our current study on astronauts who spent six months in space, offered a unique opportunity to investigate the impact of a deconditioned otolith system on blood pressure and cardiac autonomic control. In addition, it allowed us to study a temporarily hampered VSR in humans in an unprecedented way.

**Figure 2:**
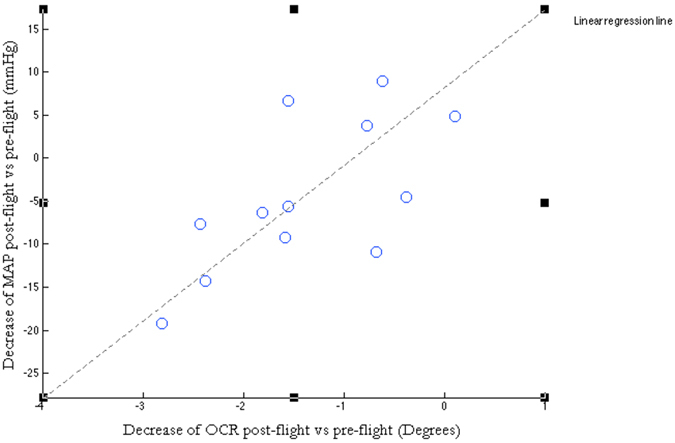
Correlation between OCR and MAP. The figure shows for each one of the 12 individuals the change in OCR (x-axis) and the change in MAP (y-axis). The change in OCR and MAP reflects the effect of long-term exposure of microgravity on the vestibular and the cardiovascular system. A statistical significant correlation between the OCR and MAP (r = 0.67, p = 0.018) was found. An individual with a decrease in OCR on return was also more likely to have a decrease in MAP (while standing). The same was true for the reverse; an astronaut with a fairly intact otolith reflex better could maintain his blood pressure upon standing. This indicates that an individual with a decreased otolith function is also more likely to have a difficulty to maintain blood pressure while standing.

**Figure 3:**
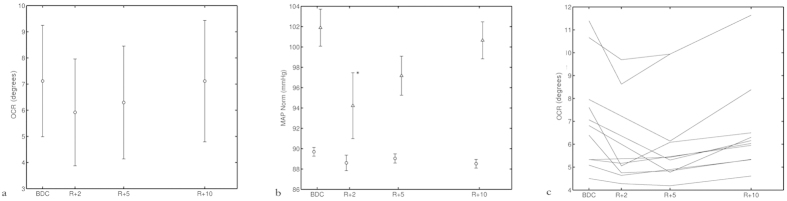
Recovery of (**a**) OCR, (**b**) MAP and (**c**) OCR per individual upon return. The figure shows the average OCR (a) and MAP (b) for the 12 subjects before and after a 6-month spaceflight. c shows the OCR recovery per individual. On the x-axis the experiments, BDC (baseline, measured before flight), R + 2, R + 5 and R + 10 are indicated. (Return + “the number of days after return the experiment was performed”). The y-axis shows the OCR in degrees and MAP in mmHg. Triangular data points represent data collected during standing position and the circular data points represents the supine position. Both variables, OCR and MAP, were significantly decreased after spaceflight (* indicates a significance of P ≤ 0.05). On the last experiment day (R + 10), they were both back at the same level as before flight (BDC), indicating a full recovery from the microgravity effect. The individual differences in OCR is in general rather big, in figure c the OCR is displayed individually for our 12 subjects. The individual decrease in response after spaceflight is clearer in c.

Comparison of pairwise preflight data with postflight data, for each astronaut individually, resulted in a statistically significant decrease of 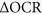 (p = 0.00076). This result agrees with earlier studies showing a decreased OCR on return after spaceflight^31^,^32^,^33^,^34^. More importantly, for the early postflight experiment there was a significant correlation between the 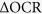, and a reduced blood pressure response; 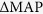 (r = 0.67, p = 0.018). (Fig. 2) MAP is represented as the average of 2/3 DBP + 1/3 SBP. We found that individuals with a decreased otolith function also had a reduced cardiovascular function on their return to Earth. Conversely, astronauts whose otolith function seemed unaffected by spaceflight also had a low or no decrease of their blood pressure control function. Hence, we demonstrate a significant one-to-one relationship between the otolith reflex OCR and the MAP. In addition, both OCR and MAP were back at baseline level at the late postflight experiment (R + 10). (Fig. 3) In all the diagrams presenting cardiovascular data, the triangular data points represent data collected during standing position and circular data points represent data collected during supine position.

**Figure 4:**
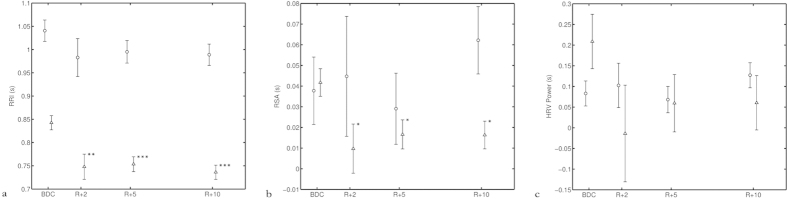
Recovery of (**a**) HR (RRI), (**b**) HF and (**c**) HRV upon return. The figure shows the results for the three parameters: HR (**a**), HF (**b**), and the total power of HRV (**c**) for the 12 subjects. On the x- axis the experiments, BDC (baseline measured before flight), R + 2, R + 5 and R + 10 are indicated. (Return + “the number of days after return the experiment was performed”). The y- axis shows for the three parameters: a; HR (RRI) in seconds (s), b; HF in seconds (s) and c; HRV in seconds (s). Triangular data points represent data collected during standing position and the circular data points represents the supine position. None of the three parameters did show a correlation with the OCR. Additionally, the HR and HF remained decreased late postflight (*p ≤ 0.05, **p ≤ 0.01 and ***p ≤ 0.001) for the standing posture. Furthermore, there was no recovery back to the preflight level during the first 10 days after return, while this was the case for OCR and MAP. No significant changes were observed in the supine posture for those parameters.

Further on, the HR (presented as RRI), HF and the total power of HRV (Fig. 4), did not show such a correlation with the OCR. Additionally, the HR and HF remained decreased late postflight (p < 0.05), while this was not the case for MAP. Again, MAP was back at preflight level at the late postflight experiment, just as the OCR. The two correlated parameters showed a similar pattern of alteration and recovery. (Figure 3)

**Figure 5:**
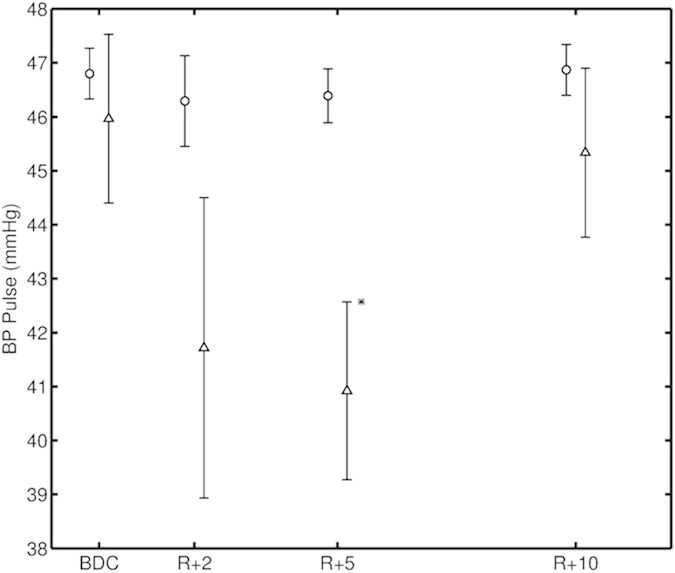
The effect of spaceflight on pulse pressure control. The figure shows the results for the blood pressure control for the 12 subjects. On the x-axis the experiments, BDC (baseline measured before flight), R + 2, R + 5 and R + 10 are indicated. (Return + “the number of days after return the experiment was performed”). The y-axis shows for the pulse pressure values in (mmHg). Triangular data points represent data collected during standing position and the circular data points represents the supine position. Pulse pressure was significantly decreased on R + 5 (*p ≤ 0.05). On R + 10, it was back at the same level as before flight (BDC), indicating a full recovery from the microgravity effect, just as for OCR. No significant changes were observed in the supine posture. A decrease was observed on R + 2, but no significance was found due to the low number of subject tested that day (n = 5).

Pulse pressure was significantly decreased (p < 0.05) on the second experiment after spaceflight. On late postflight it was back at the same level as before flight (BDC), indicating a full recovery from the microgravity effect, just as OCR. No significant changes were observed in the supine posture. A decrease is observed on the first experiment after spaceflight (R + 2), but not significant due to the low number of subject tested that day (n = 5) (Fig. 5).

## Discussion and Conclusion

We have investigated what the effect is of a defect otolith system on the ability to regulate blood pressure during a position change. Our goal has been to elucidate a hampered VSR, the link between the vestibular and cardiovascular system. That it is a suppression of the otolith inputs to the brain instead of a stimulation of the activity has been the key to our study. Other studies have so far, only pointed toward an increase of the VSR during stimulation, instead of evaluating the effect of a suppression of the vestibular system. To the best of our knowledge, this is the closest anyone has come so far to show a relation between a suppression of the otolith response and a parallel decrease of blood pressure control in humans. We are convinced that this is a unique dataset revealing the link between the otoliths and blood pressure control mechanisms. A correlation by itself is not the shear proof of causality but together with the rest of our findings it strongly suggest that there is one. In addition, our conclusion is also based on results from earlier research studies showing that a defect otolith system could affect the ability to control the blood pressure^3^.

A decreased OCR and a depression of all blood pressure and cardiac control parameters were observed during the first days after return from space. After ten days the OCR was back at the same level as before flight. This indicates that the otolith deficiency is temporary. The MAP showed the same type of recovery as the OCR (Fig. 3) after return, while on the contrary, HR and HF remained depressed. Neither were they significantly correlated with OCR at the first place. For those parameters (HR and HF) we saw that their response to orthostatic challenge remained low for all of the experiments after spaceflight, which means that on the late postflight experiment we still measured lower values than before flight. The values were not much different late postflight compared to the early postflight recording session. This indicates an independence from the vestibular activation. These findings raise evidence for a link between the otolith system and the BP regulation, namely the VSR. At the same time, most of the returning astronauts report that symptoms like dizziness and balance problems ease after a couple of days after return, which also indicating a relatively fast recovery. While this is not the case for cardiac autonomic control (HR and HF) which remains depressed but is not associated with the dizziness symptoms.

A decrease in pulse pressure could theoretically be the cause of a lower stimulation of the baroreceptors. This could in turn induce a sympathetic activation (which is what we observe), and therefore end in a better blood pressure control (maintenance of MAP). However, to explain the observed decreased MAP control, the pulse pressure data should then demonstrate an increased pulse pressure after spaceflight. An increased pulse pressure could elicit a peripheral vasodilatation, responsible for the observed decrease in MAP. But this is not what we found. On the contrary, we measured a decrease in pulse pressure after spaceflight, something that is very unlikely to explain the decreased MAP on return.

As the standing values were taken 10 minutes after the tilt, we can stress the evidence that a sympathetic activation, on early postflight, was not able to counteract the decrease in MAP. MAP still remained lower than normal. However, late postflight, we observed a similar sympathetic activation as early postflight (similar decrease in HR and in HF) but MAP returned back to normal. For the OCR we saw the same recovery pattern as for the MAP, a full recovery at late postflight. Therefore our data suggest that the return back to normal values of blood pressure control is to due the VSR. Rather than due to the central autonomic control of the cardiovascular system, which remains under a high sympathetic activation. We speculate that, when the otolith function returns back to normal, the VSR helps preventing a blood pressure drop that the activation of the sympathetic system can not do alone.

The finding that a decreased ability to maintain blood pressure upon standing is correlated with a decreased otolith response do not only has important implications for human space exploration. The acquired knowledge possibly suggests looking at OI problems also from a vestibular point of view. Currently, the cardiovascular clinical approach to OI does not consider a vestibular component, but this is understandable since only recently the evaluation of the otolith system has become more readily available to the clinicians. Nevertheless, for the future, we anticipate that the current findings may spur interdisciplinary research regarding a vestibulo-sympathetic reflex. Besides these important applications, we consider our finds to be of fundamental scientific interest, since they strengthens the evidence of a fundamental physiological otolith-sympathetic reflex (VSR) in humans.

All participants provided written informed consent prior to their participation. The study protocol was designed in accordance with the Declaration of Helsinki and was approved by the Institutional Review Board of the ESA and the Antwerp University Hospital.

## Additional Information

**How to cite this article**: Hallgren, E. *et al.* Dysfunctional vestibular system causes a blood pressure drop in astronauts returning from space. *Sci. Rep.* **5**, 17627; doi: 10.1038/srep17627 (2015).
